# The use of the CNIC-Polypill in real-life clinical practice: opportunities and challenges in patients at very high risk of atherosclerotic cardiovascular disease – expert panel meeting report

**DOI:** 10.1186/s12919-023-00268-9

**Published:** 2023-08-17

**Authors:** Lilian Grigorian-Shamagian, Antonio Coca, Joao Morais, Pablo Perez-Martinez, the Multinational Discussion Group, Adriana Barragan, Ana Isabel Barrientos, Alexandre Amaral e Silva, Akhmetzhan Sugraliyev, Alexander Parkhomenko, Álvaro Sosa Liprandi, Biljana Parapid, Carlos Olivares, Carlos Ignacio Ponte Negretti, Daniel Quesada, Dragana Kosevic, Edith Ruiz Gastelum, Emilio Samael Peralta López, Francisco Araujo, Francisco Gerardo Padilla Padilla, François Krzesinski, Imad Alhaddad, Jose Alejandro Chavez Fernandez, Jose R. Gonzalez-Juanatey, M. Samir Arnaout, Mar Castellanos, Maxima Mendez, Monica Acevedo, Olena Koval, Pablo Jorge, Parounak Zelveian, Reinhold Kreutz, Vira Tseluyko

**Affiliations:** 1 Department of Cardiology, Hospital General Universitario Gregorio Marañón, Instituto de Investigación Sanitaria Gregorio Marañón and Facultad de Medicina, Universidad Complutense de Madrid, Madrid, Spain; 2 Centro de Investigación Biomédica en Red en Enfermedades Cardiovasculares (CIBERCV), Instituto de Salud Carlos III, Madrid, Spain; 3 Hypertension and Vascular Risk Unit, Department of Internal Medicine, Hospital Clínic, University of Barcelona, Barcelona, Spain; 4 Leiria Hospital Centre, Leiria, Portugal; 5 ciTechCare - Center for Innovative Care and Health Technology, Polytechnique of Leiria, Leiria, Portugal; 6 Lipids and Atherosclerosis Unit, IMIBIC/Reina Sofia University Hospital/University of Córdoba, Córdoba, Spain; 7 CIBER Fisiopatologia Obesidad Y Nutricion (CIBEROBN), Instituto de Salud Carlos III, Madrid, Spain

**Keywords:** Polypill, Fixed-dose combination, Secondary prevention, Cardiovascular disease, Cerebrovascular disease

## Abstract

Although the cardiovascular (CV) polypill concept is not new and several guidelines state that a CV polypill should be considered an integral part of a comprehensive CV disease (CVD) prevention strategy, there are still some barriers to its implementation in the real-world setting, mainly in secondary CV prevention. As the CNIC-polypill is the only one approved for secondary CV prevention in patients with atherosclerotic CVD in 27 countries worldwide, a panel of four discussants and 30 participants from 18 countries conveyed in a virtual meeting on April 21, 2022, to discuss key clinical questions regarding the practical use of the CNIC-Polypill and barriers to its implementation.

Data presented showed that, although the use of the CV polypill is not explicitly mentioned in the current 2021 European Society of Cardiology guidelines on CVD prevention, it may be used in any patient for secondary CVD prevention tolerating all their components to improve outcomes through different aspects. The favourable results of the Secondary Prevention of Cardiovascular Disease in the Elderly (SECURE) trial now reinforce this recommendation. The panellists presented algorithms on how to switch from any baseline regimen when starting treatment with the CNIC-polypill in different situations, including patients with hypertension, dyslipidaemia, and a previous CV event; at discharge after a cardiovascular event; in chronic ischemic conditions; and in cases of polypharmacy. The panellists and expert discussants did agree that available studies conducted so far with the CNIC-polypill demonstrate that it is as efficacious as the monocomponents, equipotent drugs, or other therapies; reduces the risk of experiencing recurrent major CV events; improves medication adherence; reduces health care costs and resources compared to patients treated with loose drugs; and the patients prefer it over the multipill strategy.

In conclusion, the data presented by the participants provided the evidence behind the use of the CNIC-polypill to help fulfil the goal of encouraging its adoption by physicians.

## Background

### Rationale and objectives of the expert panel meeting

The concept of the cardiovascular (CV) polypill was first proposed in 2003 as a fixed-dose combination (FDC) of generic pharmaceutical components to target different major CV risk factors [1]. In the following years, different polypills –composed of at least one antihypertensive, a lipid-lowering medication, with and without aspirin– were registered and marketed for primary and secondary CV disease (CVD) prevention, first in India in 2009 (Polycap®, Cadila) and later in several European countries in 2014 (Trinomia®, Ferrer) [2]. This new combination is particularly relevant as it brings together three of the most studied drugs in the setting of CVD prevention: aspirin, ramipril, and atorvastatin.

Based on the initial evidence-based effectiveness and safety of the CV polypill, the 2018 European Society of Cardiology (ESC) clinical guidelines on the management of acute myocardial infarction stated that a CV polypill should be considered an integral part of a comprehensive secondary CVD prevention strategy [3]. Moreover, single-pill combinations are endorsed by the 2018 European Society of Hypertension and the 2018 and 2020 updates of the American Heart Association Hypertension guidelines [4–6]. Beyond these recommendations, there is general agreement among the experts that, based on the evidence to date, showing a remarkable clinical benefit, there is a need to improve the implementation of CV polypills in the context of a global shift in treatment paradigms [2, 7, 8]. This is because CV polypills seemingly remain underutilised in clinical practice globally, which is attributed to different practical issues, including the physician’s perspective, health system barriers, and patient factors [2, 7–9]. The physician’s defiance of FDC prescription has been reported to be driven by inexperience using combination therapies, the perception that there is no established evidence base, the inability to titrate dosage when the desired therapeutic effect is not achieved (although additional drugs can be added to the base treatment with the CV polypill), the risk of adverse effects of one the components potentially leading to non-adherence to all medications, and the physician’s impression that there is a lack of universal guidelines supporting the use of CV polypills [7, 9, 10]. To face the physician’s resistance and to scale up the use of CV polypills, several approaches have been proposed: medical education campaigns, the development of pragmatic, simplified treatment and monitoring algorithms (for polypill initiation, titration, and substitution), the implementation of electronic decision tools, and the elaboration of clinical guidelines on the use of FDC endorsed by national or international key opinion leaders, CVD organisations, and health authorities [2, 7, 8].

Two articles were recently published in the context of the need to aid clinicians in simplifying the steps to switch from any prior regimen to the CV polypill [11, 12]. Both were focused on the use of the CNIC-polypill, containing a statin (atorvastatin), an antihypertensive agent (the angiotensin-converting-enzyme inhibitor [ACEI] ramipril), and aspirin [13]. We report here the summary of a virtual expert panel meeting where key opinion leaders from Spain and Portugal convened on April 21, 2022, to 1) present algorithms on how to switch to/initiate treatment with the CNIC-polypill in different clinical situations; 2) formulate and discuss answers to clinical questions regarding the use of the CNIC-polypill in real-life secondary CVD prevention and barriers to its implementation; and 3) answer and discuss open questions from participating physicians to offer an exchange of scientific and personal delivery experiences and opinions. Finally, attendees were asked to anonymously vote online on some statements to agree on the available evidence and ways to implement the CNIC-Polypill strategy in secondary prevention.

## Presentations

### Panelist: Antonio Coca, MD, PhD, Department of Internal Medicine, Hospital Clínic, University of Barcelona

Dr Coca presented the recently published algorithms to start or switch to the most appropriate CNIC-Polypill dosage based on the hypertension grade in patients at high risk or with established CVD [11]. The presentation began by emphasising that, besides lifestyle measures, the pharmacological treatment recommended by guidelines for very high-risk patients with established CVD includes lipid-lowering drugs, antihypertensive agents, and antiplatelet therapy. This baseline regimen must be complemented with other drugs depending on the associated comorbidities. Thus, the number of pills to be taken by these patients is usually high, and adherence and persistence in treatment are low.

Dr Coca stated that the CNIC-Polypill may be used in any patient for secondary CVD prevention tolerating all their components to improve outcomes through different aspects: 1) increased adherence due to the decrease in pill burden [14, 15], 2) enhanced patient’s preferences, [16], 3) break healthcare professionals therapeutic inertia [17–19], and 4) the reported synergy between components inside the polypill [20]. Switching to the CV polypill strategy implies switching from the prior treatment with ACEIs or angiotensin receptor blockers (ARBs; alone or combined with diuretics or calcium channel blockers [CCBs]) and a statin (alone or associated with ezetimibe or a proprotein convertase subtilisin-kexin type 9 inhibitor [PCSK9i]) to the components of the polypill. Besides, in clinical practice, starting the treatment with CV polypill in patients with hypertension, dyslipidaemia, and a previous CV event, implies adapting the doses of statins and antihypertensives to the patient’s blood pressure (BP) and LDL-c levels, which may vary between patients, even when showing a similar CV risk. Dr Coca stated that this is not a problem because there are six different versions of the CNIC polypill: besides the 100 mg dose of acetylsalicylic acid (ASA) in all versions, it can contain either 2.5 mg, 5 mg, or 10 mg of ramipril and either 20 mg or 40 mg of atorvastatin.

Before describing the algorithms, Dr Coca presented tables to help switching from other available drugs to equivalent effective daily doses of the CNIC-polypill components based on extensive data on their therapeutic interchangeability. Afterwards, he presented two algorithms: 1) steps to be followed in patients in secondary CVD prevention switching from any prior multiple pill treatment with ACEIs or ARBs and statins to the CNIC-polypill, and 2) an algorithm for the selection of initial doses of the CNIC-Polypill in patients with different grades of hypertension at high risk of CVD.

#### Switching from other ACEIs or ARBs to ramipril

Because all ACEIs have similar antihypertensive efficacy, safety, and degree of CV protection, patients already taking one ACEI and not experiencing side effects can be switched to another ACEI the next day at a comparable dose. Moreover, if it is well tolerated, there is no problem substituting one ARB with one ACEI at the equivalent dose (Table 1).

**Table 1:** Approximate equivalent effective daily doses between ACEIs / ARBs and ramipril

**ACEi**	**Ramipril 2.5 mg**	**Ramipril 5 mg**	**Ramipril 10 mg**
Benazepril	10 mg	20 mg	40 mg
Captopril	50 mg	100 mg	200 mg
Cilazapril	2.5 mg	5 mg	10 mg
Enalapril	10 mg	20 mg	40 mg
Fosinopril	15 mg	30 mg	60 mg
Lisinopril	10 mg	20 mg	40 mg
Moexipril	15 mg	30 mg	60 mg
Perindopril erbumine	2 mg	4 mg	8 mg
Perindopril arginine	2.5 mg	5 mg	10 mg
Quinapril	10 mg	20 mg	40 mg
Tradolapril	2 mg	4 mg	8 mg
Zofenopril	30 mg	60 mg	120 mg
**ARB II**	**Ramipril 2.5 mg**	**Ramipril 5 mg**	**Ramipril 10 mg**
Candesartan	4–8 mg	8–16 mg	16–32 mg
Eprosartan	150 mg	300 mg	600 mg
Irbesartan	75–150 mg	150 mg	300 mg
Losartan	25–50 mg	50 mg	100 mg
Olmesartan	5–10 mg	10–20 mg	20–40 mg
Telmisartan	20 mg	40 mg	80 mg
Valsartan	40–80 mg	80–160 mg	160-320^a^ mg
Azilsartan	20 mg	40 mg	80 mg

Source: Coca J Hypertens. 2020;38(10):1890–98 [11]. Reproduced with permission

*ACE* Angiotensin-converting enzyme, *ARB* Angiotensin II receptor blocker

^a^Some dosages may exceed what is commonly recommended

#### Switching from other statins to atorvastatin

In patients with tolerated sustained statin treatment, it is possible to switch from other statins to an equivalent potency dose of atorvastatin, the lipid-lowering component of the CNIC-polypill, as shown in Table 2.

**Table 2:** Approximate daily doses with effective equivalence between other statins versus atorvastatin

Statin	Atorvastatin 20 mg	Atorvastatin 40 mg
% of LDLc reduction	**43%**^a^	**49%**^a^
Lovastatin	80 mg	-
Pitavastatin	4 mg	-
Pravastatin	80 mg	-
Rosuvastatin	5 mg	10 mg
Simvastatin	40 mg	80 mg

Source: Coca J Hypertens. 2020;38(10):1890–98 [11]. Reproduced with permission

*LDLc* Low-density lipoprotein-cholesterol

^a^Based on [21]

#### Algorithm to switch from the baseline treatment to the CNIC-Polypill in secondary prevention of patients treated with multiple drugs

**Fig. 1:**
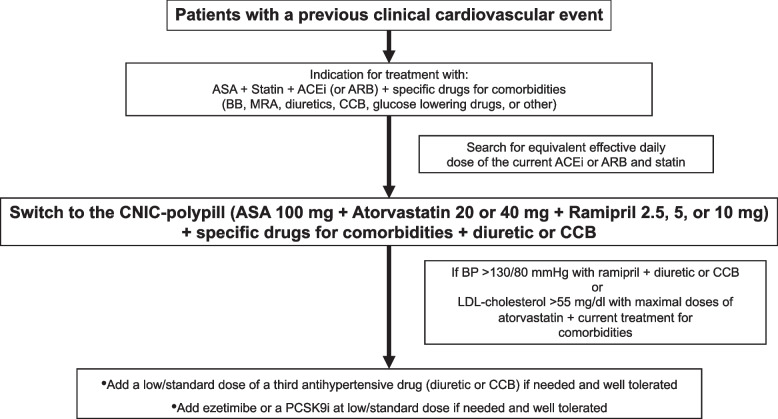
Steps for switching from baseline treatment to the CNIC-polypill in secondary prevention of cardiovascular disease in patients treated with multiple drugs/pills for their associated cardiovascular risk factors and comorbidities. ASA, acetylsalicylic acid; ACEi, angiotensin-converting enzyme inhibitor; ARB, angiotensin receptor blocker; CCB, calcium channel blocker; PCSK9i, proprotein convertase subtilisin-kexin type 9 inhibitor. Source: Coca J Hypertens. 2020;38(10):1890–98 [11]. Reproduced with permission

All patients in secondary prevention have the indication to be treated with ASA, a statin, and an ACEI or ARB. However, patients are often on other drugs to control comorbidities (e.g., beta-blockers, mineralocorticoid receptor antagonists [MRAs], diuretics, calcium channel blockers [CCBs], antidiabetics). The first step would be to find the equivalent effective doses of the current statin and ACEI/ARB to the ramipril and atorvastatin components of the CNIC polypill and add the additional drugs already taken by the patient and a diuretic or a CCB if needed (Fig. 1). In case of not achieving the strict 130/80 mm Hg BP target value with this approach, the strategy is to add a third antihypertensive (a low/standard dose of a diuretic or a CCB). If the target not achieved is the LDL-c level < 55 mg/dL with the maximum tolerated dose of statin, the approach would be to add ezetimibe and/or a PSCK9i and/or other available lipid-lowering drugs (i.e., bempedoic acid).

#### Algorithm for the selection of initial doses of the CNIC-Polypill in patients with different grades of hypertension at high risk of CVD

**Fig. 2:**
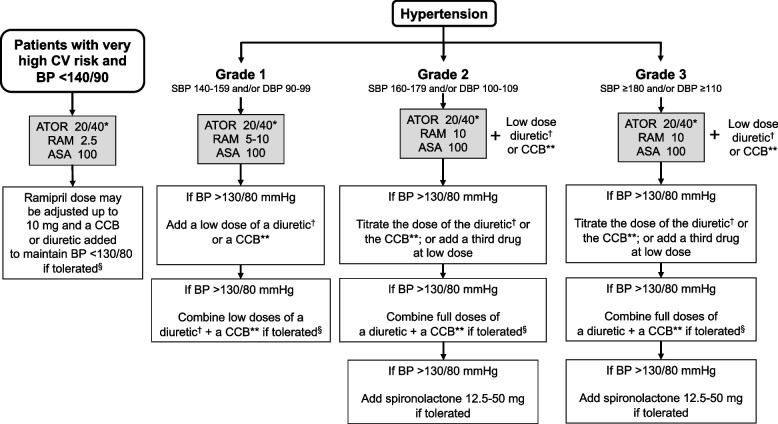
Algorithm for using the CNIC-polypill in hypertensive patients at very high risk of cardiovascular disease. AAS, acetylsalicylic acid; ATOR, atorvastatin; BP, blood pressure; CCB, calcium channel blocker; diuretic: hydrochlorothiazide, chlortalidone or indapamide; PCSK9i, proprotein convertase subtilisin-kexin type 9 inhibitor; RAM, ramipril. *Twenty or 40 mg of atorvastatin based on initial and target LDL-cholesterol. If the target is not achieved, ezetimibe/PCSK9i can be added. **Diuretic or CCB depending on patient’s metabolic profile. ^†^Low-dose: half standard dose. ^§^In patients with heart failure with reduced ejection fraction (HFrEF), the priority is to add spironolactone before using high doses of CCBs. Source: Coca J Hypertens. 2020;38(10):1890–98 [11]. Reproduced with permission

In patients with hypertension, all clinical guidelines agree that, depending on the hypertension grade, all patients must be treated from the beginning with two antihypertensive drugs [4, 22, 23]. Thus, the CNIC-polypill can be used in patients with grade 1 hypertension, but for those with grade 2 or 3 hypertension, we need to add a low dose of a CCB or a diuretic to the polypill depending on the metabolic profile of the patient (Fig. 2). When the stricter BP < 130/80 mm Hg is not achieved, the ramipril dose of CNIC-polypill ramipril can be increased to the maximum (10 mg) and increase the dose of the diuretic or CCB to the maximum. If still not at target, the three drugs (ramipril, a CCB and a diuretic) can be combined at full doses.

### Panelist: Lilian Grigorian-Shamagian, MD, PhD, Department of Cardiology, Hospital General Universitario Gregorio Marañón, Madrid

Dr Grigorian-Shamagian’s presentation focused on the recently published practical decision algorithms to facilitate the use of the CNIC-Polypill strategy for secondary CVD prevention in outpatient clinics, rehabilitation institutions, or primary care [12]. She presented three different algorithms: 1) one for secondary prevention in patients after an acute coronary syndrome (ACS); 2) one for secondary prevention in patients with chronic CVD; and 3) one to reduce polypharmacy in patients with chronic CVD and controlled blood pressure and hyperlipidaemia.

#### Algorithm for the use of the CNIC-Polypill for secondary prevention after an acute event

Dr Grigorian-Shamagian’s first described and clarified when and in which patients hospitalised for an ACS use the CNIC-polypill is appropriate. She stated that the most convenient moment to start the CNIC-polypill treatment is right at hospital discharge or soon after it or at cardiac rehabilitation clinics. At hospital admission and during the early hospital stay, the patient may experience haemodynamic instability and may require complex additional/concomitant treatment or invasive procedures, making the CNIC-polypill an unsuitable strategy. Regarding the most appropriate candidates, this is mainly linked to CNIC-polypill components. Firstly, the prescription of the CNIC-polypill does not seem appropriate to patients with indication for long-term or permanent chronic anticoagulation (e.g., with high-risk atrial fibrillation, mechanical valve, and pulmonary and/or venous thromboembolism) because aspirin needs usually to be withdrawn in the first month after the acute event. Secondly, it might not be indicated in patients with chronic symptomatic heart failure (HF) with reduced ejection fraction and an indication for an angiotensin receptor neprilysin inhibitor (ARNI), because of the interaction with the CNIC-polypill ramipril. Finally, the CNIC-polypill is inappropriate if the patient has a contraindication to any of the components.

**Fig. 3:**
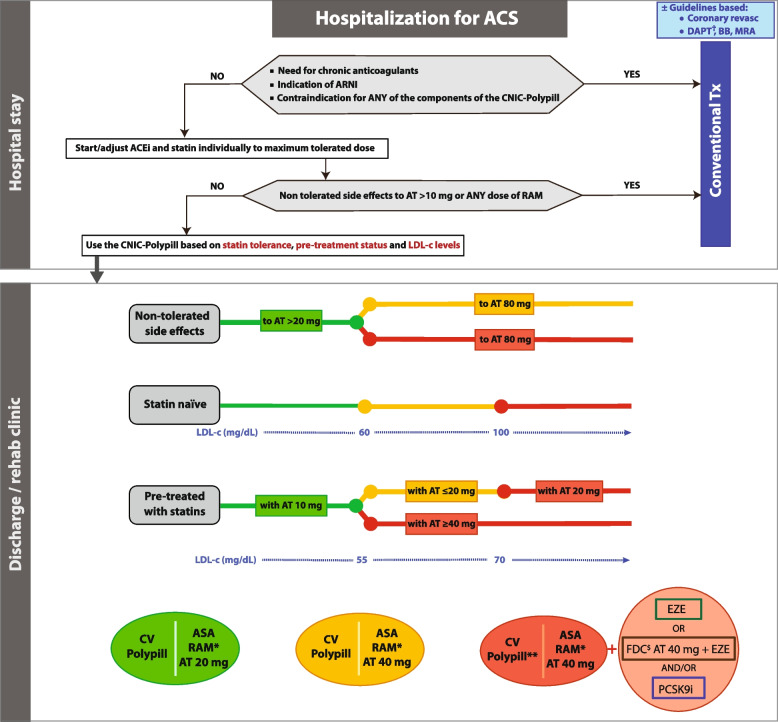
The algorithm shows the steps and options to switch patients hospitalised for an acute coronary syndrome to the CV polypill strategy. Note: The coloured balls represent the appropriate formulation of the CNIC-Polypill according to the coloured lines of the algorithm. ^†^Select P2Y12 inhibitor in addition to the CNIC-Polypill. *Dose adjusted to BP levels, previous ACEI/ARB dose and renal function. **Reassess in 3–4 weeks after discharge and readjust the dosage, consider adding A40 or ezetimibe and/or a PCSK9i. ^$^Use only if the patient does not develop side effects to atorvastatin 80 mg (or equivalent doses of another statin). ACEI, angiotensin-converting enzyme inhibitor; ACS, acute coronary syndrome; ARB, angiotensin II receptor blocker; ARNI, angiotensin receptor neprilysin inhibitor; ASA, acetylsalicylic acid; AT, atorvastatin; BB, beta blocker; BP, blood pressure; DAPT, dual platelet therapy; EZE, ezetimibe; FDC, fixed-dose combination; LDL-c, low-density lipoprotein cholesterol; PCSK9i, proprotein convertase subtilisin/kexin type 9 inhibitor; RAM, ramipril; Tx, treatment. Source: Grigorian-Shamagian et al. Front Cardiovasc Med. 2021;8:663,361 [12]. Reproduced with permission

Dr Grigorian-Shamagian’s described the algorithm for patients who experienced an ACS (Fig. 3). The first step as per clinical guidelines after an ACS is to start treatment with antiplatelet therapy and an ACEI, and a statin at the maximum tolerated doses. As the lowest dose of atorvastatin in the CNIC-polypill is 20 mg, if the patient tolerates > 10 mg of atorvastatin/or equivalent dose of another statin (the vast majority of the patients) and any dose of ramipril/or other ACEI (or equivalent dose of ARB), the CNIC-polypill strategy can be initiated at discharge based on different factors that will indicate the dose of atorvastatin and ramipril to be used: 1) for atorvastatin, depending on statin tolerance and LDL-c levels, but usually the highest dose should be used; 2) for ramipril, based on BP levels, prior ACEI/ARB dose, and kidney function; and 3) although the 100 mg ASA component of the CNIC-polypill does not need to be further adjusted, we need to add an additional antiplatelet drug (a P2Y12 inhibitor) for usually a 12 month-period.

**Fig. 4:**
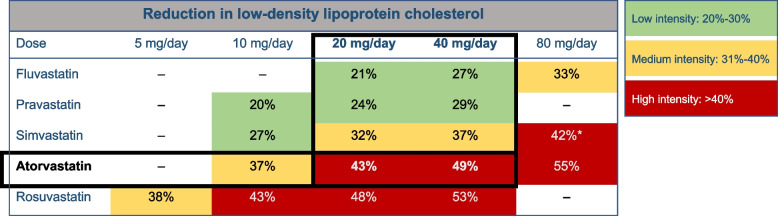
Grouping of statins by intensity categories according to the percentage reduction in low-density lipoprotein [21]. *Advice from the MHRA (Medicines and Healthcare products Regulatory Agency): there is an increased risk of myopathy associated with high-dose (80 mg) simvastatin. The 80 mg dose should be considered only in patients with severe hypercholesterolemia and high risk of cardiovascular complications who have not achieved their treatment goals on lower doses, when the benefits are expected to outweigh the potential risks

Following this introduction, Dr Grigorian-Shamagian’s described the steps to select the most adequate version of the CNIC-polypill according to statin tolerance, prescription status, and dose (Fig. 3). Briefly, if the patient develops side effects such as liver or muscular toxicity, the dose of atorvastatin (or equivalent doses of other statins) must be chosen based on the tolerated doses and add other lipid-lowering drugs (e.g., ezetimibe, PSCK9i, inclisiran and/or bempedoic acid) if the CNIC-polypill alone is not sufficient to reach the LDL-c target level. For statin-naïve patients, the dose of atorvastatin to be chosen will depend on the LDL-c levels at discharge and the expected per cent reduction with that dose, namely 43% with atorvastatin 20 mg and 49% with atorvastatin 40 mg (Fig. 4). When the target (< 55 mg/dL) is not achieved, an additional dose of 40 mg of atorvastatin or the addition of other lipid-lowering drugs to the CNIC-polypill base treatment must be considered. Finally, in patients pre-treated with statins, the version of the CNIC-polypill must be chosen based on the LDL-c level at discharge and the previous dose of the prescribed statin using the table of equivalence (Table 2): 1) the CNIC-polypill with 20 mg of atorvastatin can be used alone in a very small proportion of patients previously on atorvastatin 10 mg/equivalent dose of another statin or statin naïve, and low LDL-c levels (< 55 mg/dL) to intensify statin treatment in patients with a recent acute event; 2) the CNIC-polypill version with 40 mg of atorvastatin can be used alone in patients previously on atorvastatin ≤ 20 mg/equivalent dose of another statin and LDL-c level between 55 and 70 mg/dL); and 3) the CNIC-polypill version with 40 mg of atorvastatin is to be prescribed to patients previously on atorvastatin ≤ 20 mg/equivalent dose of another statin and LDL-levels above 70 mg/dL, with additional lipid-lowering drugs. Patients experiencing an ACS must be monitored and re-evaluated in 4–8 weeks in the outpatient setting.

#### Algorithm for the use of the CNIC-Polypill for secondary prevention in chronic CVD

Dr Grigorian-Shamagian described the algorithm to be used by clinicians who want to use the CNIC-Polypill in patients with chronic conditions, defined as a chronic coronary syndrome (CCS), established atherothrombotic stroke, or peripheral artery disease (PAD). Alleged medical conditions, side effects or intolerances preventing the use of the CNIC-polypill strategy have been previously discarded. These include high bleeding risk, statins tolerance, presence of symptomatic HF with reduced ejection fraction, haemorrhagic stroke, and patients with severely impaired renal function, whose treatment with ACEIs should be closely monitored.

**Fig. 5:**
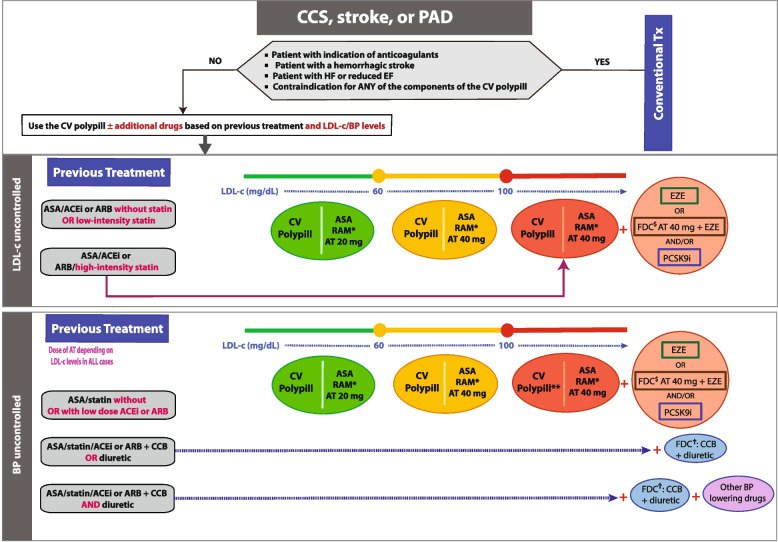
The algorithm shows the steps and options to switch patients treated for chronic coronary syndromes to the CNIC-Polypill strategy. Note: The coloured balls represent the appropriate formulation of the CNIC-Polypill according to the coloured lines of the algorithm. *Dose adjusted to BP levels, previous ACEI/ARB dose and renal function. **See above for additional treatment of high LDL-c levels. ^$^Use only if the patient does not develop side effects to atorvastatin 80 mg (or equivalent doses of another statin). ^†^If the combination is available. ACEI, angiotensin-converting enzyme inhibitor; ARB, angiotensin II receptor blocker; ASA, acetylsalicylic acid; AT, atorvastatin; BP, blood pressure; CCB, calcium channel blocker; CCS, chronic coronary syndrome; EZE, ezetimibe; FDC, fixed-dose combination; LDL-c, low-density lipoprotein cholesterol; PAD, peripheral artery disease; PCSK9i, proprotein convertase subtilisin/kexin type 9 inhibitor; RAM, ramipril; Tx, treatment. Source: Grigorian-Shamagian et al. Front Cardiovasc Med. 2021;8:663,361 [12]. Reproduced with permission

For patients with chronic conditions, the algorithm is based on the previous pharmacological treatment and whether the patient’s BP and LDL-c are well-controlled, thus requiring, in certain circumstances, the addition of concomitant drugs (Fig. 5). Briefly, when LDL-c levels are suboptimally controlled (> 55 mg/dL), and depending on the previous statin potency, we can always switch to the CNIC-polypill version with 20 or 40 mg depending on the LDL-c level, and in some patients consider adding other lipid-lowering drugs. Regarding patients with uncontrolled BP, the ramipril dose to be chosen from the available CNIC-polypill versions (i.e., 2.5, 5, or 10 mg) will depend on the hypertension grade and based on the equivalent effective daily doses, as described by Dr Coca (Table 1). Moreover, additional BP-lowering drugs may be added to the base treatment in resistant cases.

#### Algorithm to reduce polypharmacy in patients with established CVD, well-controlled blood pressure and low-density lipoprotein cholesterol, and treated with 5 or more drugs

**Fig. 6:**
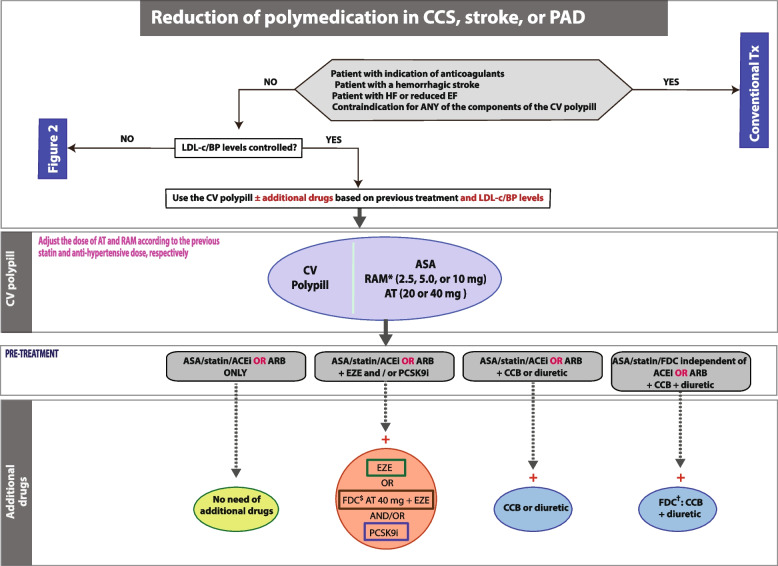
The algorithm shows the steps and scenarios to reduce the pill burden with the CNIC-Polypill in patients treated for chronic CVD. Note: *Dose adjusted to previous ACEI/ARB dose and renal function. $Use only if the patient does not develop side effects to atorvastatin 80 mg (or equivalent doses of another statin).^†^If the combination is available. ACEI, angiotensin-converting enzyme inhibitor; ARB, angiotensin II receptor blocker; ASA, acetylsalicylic acid; AT, atorvastatin; BP, blood pressure; CCB, calcium channel blocker; CCS, chronic coronary syndrome; EZE, ezetimibe; FDC, fixed-dose combination; LDL-c, low-density lipoprotein cholesterol; PAD, peripheral artery disease; PCSK9i, proprotein convertase subtilisin/kexin type 9 inhibitor; RAM, ramipril; Tx, treatment. Source: Grigorian-Shamagian et al. Front Cardiovasc Med. 2021;8:663,361 [12]. Reproduced with permission

Dr Grigorian-Shamagian stressed the need to reduce the number of pills in patients with chronic conditions that are well controlled and pointed at the CNIC-polypill strategy as convenient to increase adherence and eventually reduce the recurrence of CV events and related costs. She started the description of this algorithm summarising that, in patients well controlled, there is no need for additional drugs to the base treatment with the CNIC-polypill and thus we only need to adjust the doses of the polypill components to the previous doses that allowed optimal LDL-c and BP levels. Based on the proposed algorithm (Fig. 6), the CNIC-polypill strategy will allow prescribing only one pill instead of three in patients on ASA, a statin, and an ACEI or ARB. In other cases, we can reduce the CNIC-polypill with other fixed-dose combinations from 3, 4, or 5 pills to 2 pills.

**Fig. 7:**
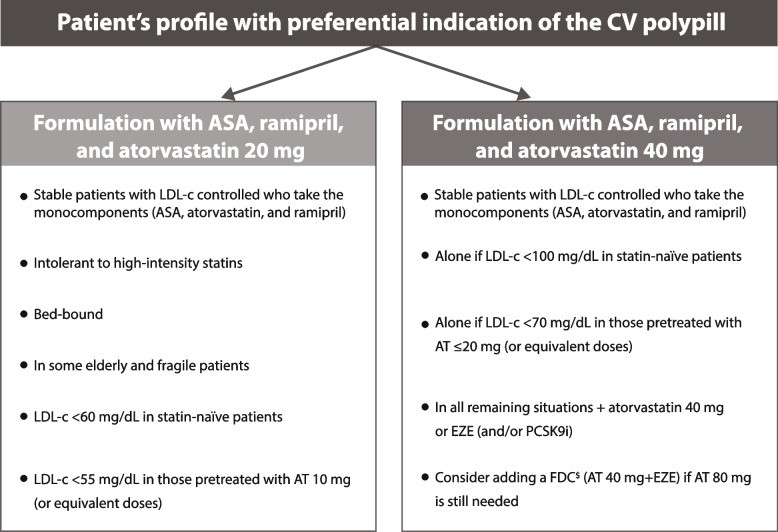
Summary of the adequate profile of patients considered candidates to switch to the CNIC-Polypill. Note: *Or equivalent doses of the monocomponents. ^$^Use only if the patient does not develop side effects to atorvastatin 80 mg (or equivalent doses of another statin). ACS, acute coronary syndrome; AT, atorvastatin; CV, cardiovascular; EZE, ezetimibe; FDC, fixed-dose combination; LDL-c, low-density lipoprotein cholesterol; PCSK9i, proprotein convertase subtilisin/kexin type 9 inhibitor. Source: Adapted from Grigorian-Shamagian et al. Front Cardiovasc Med. 2021;8:663,361 [12] with permission

Dr Grigorian-Shamagian ended the presentation by summarising the groups of patients whose profile is considered optimal to be treated with the CNIC-polypill with 20 or 40 mg of atorvastatin (Fig. 7).

## Key clinical questions

**Fig. 8:**
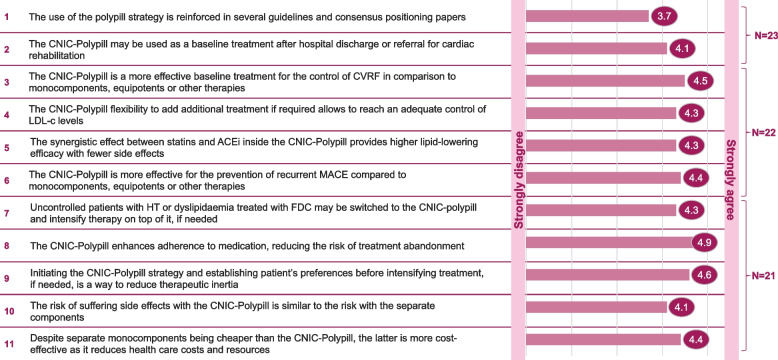
Summary of the survey results for each of the clinical statements voted by the participating physicians. ACEi, angiotensin-converting enzyme; CVRF, Cardiovascular risk factors; FDC, fixed-dose combination; HT, hypertension; LDL-c, low density lipoprotein cholesterol; MACE, major adverse cardiovascular event

Immediately after the presentations, the panellists and discussants debated a series of clinical questions focused on using the CNIC-polypill in real-life secondary CVD prevention and barriers to its implementation. These discussions were led by Dr Morais as the moderator and debated by the expert panel. After every discussed statement, all participants were asked to rate on a 5-point scale (1 = strongly disagree, 5 = strongly agree) their strength of agreement with the statements. The different statement’s discussion and the survey results for each question are summarised below and in Fig. 8.

### Clinical question 1

#### The polypill strategy is not specifically recommended in the 2021 ESC Guidelines on cardiovascular disease prevention

After this statement of Dr Morais, Dr Coca commented that the CV polypill strategy is recommended due to its increase in adherence and simplification of therapy in several guidelines [3, 4, 24] and consensus and position papers [25–28]. Although he agreed that the CV polypill is not explicitly mentioned in the 2021 ESC Guidelines on cardiovascular disease prevention [29], Dr Coca explained that when looking at the recommended strategy, the guideline clearly states that patients with established ASCVD need to start with at least statins, antihypertensive drugs, and antiplatelet therapy to reach step 1 goals (i.e., LDL-c < 70 mg/dL and SBP < 140 mmHg) [29]. Thus, despite the lack of specific recommendations, at the practical level, there is a suggestion to use these three monocomponents in only one pill to improve adherence. Moreover, the advice is to consider aiming at lower goals (step 2) once the initial goals have been achieved through the treatment intensification based on residual 10-year and lifetime CVD risk, comorbidities and degree of frailty, and patient preferences [29]. In practice, this would mean adding other drugs to the baseline treatment with the CV polypill if needed, and, in Dr Coca's opinion, the guideline's writing implicitly assumes that there is a need to reduce the number of pills to boost persistence and compliance.

### Post-discussion survey

**Statement #1: the use of the polypill strategy is reinforced in several guidelines and consensus positioning papers.** The average degree of agreement among participants to this statement was 3.7, thus between neither agreement/ nor disagreement and agreement.

**Statement #2: the CNIC-Polypill may be used as a baseline treatment after hospital discharge or referral for cardiac rehabilitation. **The average degree of agreement among participants to this question was 4.1, thus between agreement and strong agreement.

### Clinical question 2

#### Does the CNIC-Polypill provide a better CVRF management compared with monocomponents?

Dr Morais raised this question, highlighting that it is frequently discussed among clinicians whether two components in the same pill (particularly in the hypertension field) have the same efficacy as separate pills. Dr Perez contributed to the discussion presenting data from the recent retrospective, non-interventional NEPTUNO study in patients in secondary prevention [30]. In this study, the lipid profile and BP improvements with the CNIC-polypill were compared to these of the same separate monocomponents, equipotent drugs, or other therapies (1,614 patients in each cohort). After 2 years of follow-up, there was a significantly greater reduction in the absolute mean levels of all the analysed lipidic variables and blood pressure in the CNIC-polypill cohort compared with the three control cohorts (Table 3). Moreover, the increase in the proportion of patients with CV risk factors controlled (i.e., LDL-c < 70 mg/dL, triglycerides < 150 mg/dL, and SBP/DBP < 130/80 mmHg) was also higher in patients treated with the CNIC-polypill approach compared with the other treatment strategies (Table 3).

**Table 3:** Evolution of lipid parameters and blood pressure after 2 years of follow-up in the NEPTUNO study [30]

	**CNIC-Polypill** **(*n* = 1614)**	**Monocomponents** **(*n* = 1614)**	**Equipotent** **(*n* = 1614)**	**Other Therapies** **(*n* = 1614)**
**Difference between baseline and study end**				
**Lipid profile, mg/dL, mean (SD)**				
Total cholesterol,	–54.9 (43.2)*	–42.8 (45.2)* †	–31.7 (43.3)* †	–31.7 (42.4)* †
LDL cholesterol,	–19.6 (38.2)*	–12.9 (42.2)* ‡	–12.3 (39.7)* †	–9.1 (41.2)* †
HDL cholesterol,	6.5 (10.2)*	4.6 (10.5)* †	3.8 (11.0)*‡	2.8 (11.0)*
Triglycerides	–67.5 (98.7)*	–59.9 (80.3)* ‡	–56.1 (77.1)* †	–54.4 (79.5)* †
**Blood pressure, mm Hg, mean (SD)**				
SBP	–14.1 (24.8)*	–11.7 (23.9)* ‡	–10.4 (24.3)* †	–10.4 (23.6)* †
DBP	–4.5 (13.3)*	–2.5 (12.0)* †	–2.1 (12.4)* †	–1.2 (12.7)* †
**Patients with CVRF control at the study end, %**				
LDL-c < 70 mg/dL	15.4*	12.5* †	12.8* †	11.6 †
Triglycerides < 150 mg/dL	50.9*	43.4* †	42.3* †	40.7* †
SBP < 130/80 mm Hg	44.1*	37.9*, **	34.6‡	32.4‡

Source: Adapted from Gonzalez-Juanatey. Int J Cardiol. 2022;361:116–123 [30]. Reproduced with permission

*DBP* Diastolic blood pressure, *HDL* High-density lipoprotein, *LDL* Low-density lipoprotein, *SBP* Systolic blood pressure, *SD* Standard deviation

^*^p < 0.001 vs baseline

^†^*p* < 0.001; ‡*p* < 0.01; and **p < 0.05 change vs reference cohort: CNIC-Polypill

Dr Perez also summarised the results of an individual data-based meta-analysis including 3140 participants in three randomised clinical trials (RCT) (76% of whom with established CV) that were either treated as per standard of care or with 2 different versions of a CV polypill manufactured in India (CV polypill 1: aspirin 75 mg/simvastatin 40 mg/lisinopril 10 mg/atenolol 50 mg; CV polypill 2: aspirin 75 mg/simvastatin 40 mg/lisinopril 10 mg/hydrochlorothiazide 12.5 mg) [31]. After 12 months of follow-up, more patients treated with the CV polypill approach achieved the 2016 ESC recommended BP targets (62% vs 58%; risk ratio [RR] = 1.08; 95% CI = 1.02–1.15) and LDL-c goals (39% vs 34%. RR = 1.13. 95% CI = 1.02–1.25) compared with those receiving usual care.

### Post-discussion survey

**Statement #3: the CNIC-Polypill is a more effective baseline treatment for the control of CVRF in comparison to monocomponents, equipotent drugs, or other therapies.** The average degree of agreement among participants to this statement was 4.5, thus between agreement and strong agreement.

### Clinical question 3

#### Is the CNIC-Polypill an option for those patients who need higher doses of statins to control their LDL-c levels?

Dr Morais commented that the maximum dose of atorvastatin available with the CNIC-polypill is 40 mg, while clinical guidelines recommend, particularly in patients after an acute event, higher doses of statins (e.g., atorvastatin 80 mg). He asked whether this could be a barrier to using the CNIC-polypill strategy. Dr Grigorian-Shamagian answered that this is indeed considered a barrier, especially by cardiologists and Western European cardiologists, who use to prescribe high potency statins (e.g., atorvastatin 80 mg or high-dose rosuvastatin). She clarified that the potency of statins is defined by their expected reduction of LDL-c cholesterol levels, and a high potency statin can reduce LDL-c by approximately 50%. Based on the NICE guidance on lipid modification [21], atorvastatin 40 mg provides a 49% reduction in LDL-c levels (Fig. 4). This percentage could be strictly considered a high statin potency according to the 2021 ESC prevention guidelines [29], and this decrease might allow reaching the final goal of LDL-c < 55 mg/dL in many patients with an acute event. Therefore, in patients with LDL-c levels not too high during the hospital stay (e.g., < 100 mg/dL), there should not be concerns about using only 40 mg of atorvastatin. Moreover, she commented on the results of a recent pharmacodynamic study reporting that the CNIC polypill decreased the LDL-c levels by 7% more than the same dose of atorvastatin alone, suggesting a synergistic effect between statins and the ACEI components of the CNIC-Polypill [20]. Therefore, she concluded that, in many patients, the CNIC-Polypill with 40 mg atorvastatin should be enough to control LDL-c levels, and in the case that this cut-off is not reached, the CNIC Polypill provides flexibility, as ezetimibe or a fixed dose of atorvastatin 40 mg plus ezetimibe or PCSK9 inhibitors can be added to the baseline polypill treatment.

### Post-discussion survey

**Statement #4: The CNIC-Polypill flexibility to add additional treatment if required allows to reach an adequate control of LDL-c levels. **The average degree of agreement among participants to this statement was 4.3, thus between agreement and strong agreement.

**Statement #5: The synergistic effect between statins and ACEi inside the CNIC-Polypill provides higher lipid-lowering efficacy with fewer side effects. **The average degree of agreement among participants to this statement was 4.3, thus between agreement and strong agreement.

### Clinical question 4

#### Does the CNIC-Polypill reduce the recurrence of CV events?

Dr Morais raised the issue that cardiologists are usually worried about the ability of medications to reduce the recurrence of CV events when implementing a new prevention strategy. Thus, their decision to use a particular drug often relies on whether clinical trials have proven that it does reduce CV events. To discuss this issue, Dr Perez mentioned two available studies assessing the recurrence of CV events in patients treated with the CNIC polypill: the NEPTUNO study [30], already published, and another study (Secondary Prevention of Cardiovascular Disease in the Elderly [SECURE] trial; please note that at the time of the meeting in April 2022, the favourable results of the trial had not been published yet) [32].

The retrospective non-interventional NEPTUNO study used a medical records database with 6,456 patients in secondary prevention [30]. The study assessed the risk of experiencing recurrent major cardiovascular events (MACE), including coronary heart disease (acute myocardial infarction and stable/unstable angina), cerebrovascular disease (ischaemic stroke and transient ischaemic attack), PAD (intermittent claudication, ischaemia, and amputation). The results showed that, after 2 years of follow-up, the risk of MACE was higher in the identical monocomponents, equipotent medications, and other therapies cohorts than in the CNIC-polypill cohort (Hazard ratio 1.22, 1.25, and 1.27, respectively).

The SECURE study recruited 2,500 patients older than 65 with a recent myocardial infarction across seven European countries [32]. This trial compared the occurrence of MACE (defined as cardiovascular death, non-fatal myocardial infarction, non-fatal ischemic stroke, and urgent revascularisation) between the CNIC Polypill and usual care during a follow-up of 4 years as the primary outcome.

Dr Perez concluded that there is real-world evidence on the effectiveness of the CNIC-Polypill in reducing CV events.

### Post-discussion survey

**Statement #6: The CNIC-Polypill is a more effective baseline treatment for the control of CVRF in comparison to monocomponents, equipotent medications, or other therapies.** The average degree of agreement among participants to this statement was 4.4, thus between agreement and strong agreement.

### Clinical question 5

#### How would you switch from the fixed-dose combination for hypertension or dyslipidemia to the CNIC-Polypill?

Dr Morais stated that many patients with hypertension or dyslipidemia use fixed-dose combinations. This is important because different guidelines recommend starting antihypertensive treatment with FDC, and asked Dr Perez-Martinez whether there is a rule on switching from FDC to the CNIC-polypill strategy. Dr Perez-Martinez described two different scenarios where this can be envisaged. In well-controlled patients taking FDC for hypertension and/or dyslipidemia, the inconvenience of having to separate all the components makes the implementation of the polypill strategy more inconvenient. In uncontrolled patients, which is a common scenario in clinical practice, the polypill strategy could be a great opportunity. In this case, instead of insisting on treating the different CV risk factors separately, the change to the polypill strategy could provide a baseline therapy on top of which to construct the treatment, intensifying therapy as needed. Moreover, additional medications may be withdrawn once some of the drugs are not needed anymore. For instance, in patients who adhere to lifestyle modifications, some drugs may be removed in the mid-long term while maintaining the baseline therapy with the CNIC-polypill.

### Post-discussion survey

**Statement #7: Uncontrolled patients with HT or dyslipidaemia treated with FDC may be switched to the CNIC-polypill and intensify therapy on top of it, if needed. **The average degree of agreement among participants to this statement was 4.3, thus between agreement and strong agreement.

### Clinical question 6

#### If a patient continues not being adherent after switching to the polypill, he would have no treatment at all. How could you explain that?

Dr Morais commented that despite the effectiveness and increased adherence of the polypill approach, some patients are still non-adherent to even simple regimens. Dr Perez-Martinez agreed that this is indeed a possibility, but the evidence from the FOCUS RCT showed that the CNIC-polypill enhanced adherence by about a 10% compared with the same separate drugs [33]. This has been confirmed in recently published retrospective studies, such as the START and the NEPTUNO [30, 34]. Finally, the AURORA study showed a higher patient preference for the CNIC-Polypill than for monotherapies [16]. Dr Perez-Martinez emphasised the need to negotiate the regimen’s preference of the patients.

Post-discussion survey

### Post-discussion survey

**Statement #8: The CNIC-Polypill enhances adherence to medication, reducing the risk of treatment abandonment. **The average degree of agreement among participants to this statement was 4.9; thus, participants were almost in strong agreement.

### Clinical question 7

#### How do you believe the use of the CNIC-Polypill could impact therapeutic inertia?

Dr Morais mentioned the important topic of the health professional’s therapeutic inertia, a problem besides the already known drawback of patient’s non-adherence. Dr Coca added that many clinicians, despite knowing the content and recommendations from clinical guidelines, are not applying them for many complex reasons. For instance, the EUROASPIRE surveys revealed that 50% or less of patients with a myocardial infarction are not receiving the optimal strategy after discharge [35]. Dr Coca said that by facilitating or simplifying treatment for clinicians, they would assume that it is easy to prescribe only the polypill with the three optimal medications at discharge this may help not only therapeutic inertia but adherence. The latter is important if we consider that the patient’s preference is a polypill vs monocomponents, as shown by the AURORA study [16]. Overall, Dr Coca concluded that the use of the polypill may indeed help prevent or reduce therapeutic inertia.

### Post-discussion survey

**Statement #9: Initiating the CNIC-Polypill strategy and establishing patient’s preferences before intensifying treatment, if needed, is a way to reduce therapeutic inertia. **The average degree of agreement among participants to this statement was 4.6, thus between agreement and strong agreement.

### Clinical question 8

#### If there is a side effect, you need to withdraw all the treatment

Dr Morais raised the issue that any of the components of the CNIC-polypill may be associated with side effects. Dr Perez-Martinez highlighted that the FOCUS study reported that patients treated with the CNIC-Polypill report a similar proportion of adverse events as when treated with the separate components, with treatment discontinued by 4% of patients in each arm [33]. Dr Perez-Martinez stated that, in his opinion, the side effects of the CNIC-polypill are not a problem in clinical practice: firstly, and as shown by Dr Grigorian-Shamagian during her presentation, we must assess in which patients the polypill is indicated; secondly, based on his experience with the use of the CNIC-polypill during the last year, its side effects were not a big issue vs monocomponents.

### Post-discussion survey

**Statement #10: The risk of suffering side effects with the CNIC-Polypill is similar to the risk with the separate components. **The average degree of agreement among participants to this statement was 4.1, thus closer to agreement than to strong agreement.

### Clinical question 9

#### Is the CV-Polypill a cost-effective strategy to implement?

Dr Morais commented that cost-effectiveness is always an issue, although the CNIC-polypill is not an expensive regimen. Dr Perez-Martinez agreed that the drug cost is important and summarised the results of models developed last year, some of them based on the increased adherence to therapy [36, 37], while others evaluated the cost-effectiveness based on the improvement in the control of CV risk factors [38, 39]. Models based on improved adherence showed that the CNIC-polypill is a cost-effective strategy to prevent fatal and non-fatal CV events [36, 37]. More recently, the NEPTUNO study, conducted in Spain [38], demonstrated that the CNIC-polypill reduces health care costs and resources compared to patients treated with loose drugs, even after correcting for different covariates, with a reduction in total costs per patient/year of approximately 1,000€. Finally, a study conducted with a subgroup of patients from the NEPTUNO study in Portugal showed that the CNIC-polypill is a cost-effective strategy in men and women for the secondary prevention of CV disease, with more quality-adjusted life-year (QALY) gained compared to usual care with monocomponents [39].

### Post-discussion survey

**Statement #11: Despite separate monocomponents being cheaper than the CNIC-Polypill, the latter is more cost-effective as it reduces health care costs and resources. **The average degree of agreement among participants to this statement was 4.4, thus between agreement and strong agreement.

## Experts’ responses to open questions/comments

In the following section, we summarise the received open questions/comments and responses of the experts during the presentations and discussions on key clinical questions:

1. “Additional barriers are the lack of time and of work in a well-trained team. Many physicians work alone, which leads to inertia, while working in a team gives more time for education and discussion of all related to the disease.”

Dr Morais and Dr Coca agreed that this is an important point, particularly in countries where family doctors in the primary care setting tend to work in an isolated manner without the support of the team (e.g., Latin America), making it easier to tend towards therapeutic inertia. Conversely, it is easier for doctors working in the hospital setting to implement guidelines since frequent internal meetings allow discussion. For this reason, there is a need for proactive in terms of medical education to consider that primary care doctors are not working in a team. Dr Coca commented that in countries such as Spain, a model that reinforces daily meetings among physicians in primary care is being implemented to boost positive interactions.

2. “From the clinical practice perspective, at my centre CNIC-Polypill is being prescribed at discharge after bypass surgery, obtaining significantly better control of hypertension and lipid status compared to patients taking monocomponents.”

Dr Grigorian-Shamagian commented that patients discharged after bypass surgery are usually patients with chronic coronary syndromes, making it very easy to start with the CNIC-Polypill.

3. “Many of the patients we treat are elderly, and the guidelines for the geriatric population do not always coincide with the standard general guidelines. For instance, they usually recommend lower doses of certain medications, including statins.”

Dr Grigorian-Shamagian emphasised that especially in elderly, fragile patients are more liable to experience any adverse event. In these cases, atorvastatin starting doses of 20 mg or 40 mg are optimal models of treatment to reduce lipidaemia.

4. “Have any countries already adapted the CNIC-Polypill as a drug in their public health system in the protocols after a CV event?”

Dr Coca replied that in Europe and Mexico, the CV polypill is included in the public health system and the protocols after myocardial infarction but that he’s not aware of other countries where it has been implemented.

5. “The main barrier to the implementation of the CV polypill seems to be that its use is not currently in the ESC guidelines” (several participants comment).

The panellists agreed that there is evidence from the recent NEPTUNO study on the effectiveness of the CNIC-polypill in reducing CV events in secondary prevention [30]. Moreover, the results of the SECURE trial [32], published after the meeting, provided additional data that patients treated with the CNIC-polypill for 6 months after a myocardial infarction had a significantly lower risk of MACE than those on usual care. They also agreed that, in current clinical practice, physicians use off-label drugs when they are convinced that the current evidence is enough to reduce morbimortality in their patients, sometimes before the results of a particular trial are published. However, they also commented that, in some countries, the health authorities are very strict with this approach, particularly regarding reimbursement of drugs not included in guidelines or officially recommended, which is a practical barrier. Despite the drawback of the 2021 ESC CV prevention guidelines, they highlighted that there are guidelines from the ESC that did recommend its use, such as the 2017 ESC Guidelines for managing acute myocardial infarction in patients presenting with ST-segment elevation [3]. The panellists concurred that physicians must follow the guidelines, but every patient needs to be evaluated individually. Finally, although not commented during the meeting, the results of the recently published SECURE trial are expected to change the next guidelines recommendation regarding the use of CNIC-polypill in secondary prevention.

6. “Is there a scenario for using the CNIC-polypill in primary prevention?”

The panellists agreed that this is an important issue because there is no indication for using the CNIC-polypill in primary prevention. However, they acknowledged several scenarios in primary prevention where its use would be beneficial. For instance, the 2018 ESC/ESH guidelines for the management of arterial hypertension [4], and the consensus document of the Spanish Society of Cardiology [27], suggest the use of the CV polypill as a substitution therapy in patients in which it is indicated the use of a statin, ACEi or ARB and aspirin. This happens in patients with advanced atherosclerosis and at high risk of a CV event, and it is particularly common in patients with diabetes. In this line, the American Diabetes Association (ADA) recommends using aspirin as a primary prevention strategy in those with diabetes who are at increased CV risk [40]. Despite not being recommended by the ESC guidelines, actually many cardiologists prescribe aspirin for primary prevention. In this scenario, the CNIC-polypill could be beneficial and would reduce the number of patients with high CV risk [41]. In this study, they already identified type 2 diabetes patients with hypertension as a patient profile that benefits from using the CNIC-Polypill in primary prevention.

## Implications for clinical practice / conclusion

In conclusion, the virtual meeting was an opportunity to understand the challenges and opportunities for advancing the CV polypill strategy in secondary prevention of CVD in current clinical practice. The opinions, discussions, and data presented by the participants of the expert panel meeting provided the evidence behind the use of the CNIC-polypill to help fulfil the goal of encouraging its adoption by physicians. As discussed in this meeting, implementation principles could lead to a behaviour change among cardiologists, primary care physicians, or other specialists (e.g., neurologists, internal medicine specialists, or endocrinologists), enhancing the opportunity for goal attainment, overcoming therapeutic inertia, and promoting medication adherence. Based on the positive participant evaluation of this interactive approach, we hope to undertake further events with a similar structure, where physicians can directly ask questions and share learning in real-time with experts in the field regarding the CV polypill strategy.

Based on all the scientific evidence obtained in CV secondary prevention regarding the effect on major outcomes the CNIC polypill strategy should be considered as baseline therapy in post-event patients. We therefore expect that the CNIC-polypill will appear as highly recommended in upcoming clinical guidelines.

## Data Availability

Not applicable.

## Abbreviations

ACEI: Angiotensin-converting enzyme inhibitor
ACS: Acute coronary syndrome
ARB: Angiotensin receptor blocker
ARNI: Angiotensin receptor neprilysin inhibitor
ASA: Acetylsalicylic acid
ASCVD: Atherosclerotic cardiovascular disease
BP: Blood pressure
CCB: Calcium channel blocker
CCS: Chronic coronary syndrome
CV: Cardiovascular
CVRF: Cardiovascular risk factor
DBP: Diastolic blood pressure
ESC: European Society of Cardiology
FDC: Fixed-dose combination
HDL-c: High-density lipoprotein cholesterol
LDL-c: Low-density lipoprotein cholesterol
MACE: Major adverse cardiovascular event
PAD: Peripheral artery disease
PCSK9i: Proprotein convertase subtilisin-kexin type 9 inhibitor
RCT: Randomised clinical trial
SBP: Systolic blood pressure
