# Correction: The use of the CNIC-Polypill in real-life clinical practice: opportunities and challenges in patients at very high risk of atherosclerotic cardiovascular disease – expert panel meeting report

**DOI:** 10.1186/s12919-023-00279-6

**Published:** 2023-10-12

**Authors:** Lilian Grigorian-Shamagian, Antonio Coca, Joao Morais, Pablo Perez-Martinez, the Multinational Discussion Group

**Affiliations:** 1 Department of Cardiology, Hospital General Universitario Gregorio MarañónInstituto de Investigación Sanitaria Gregorio Marañón and Facultad de MedicinaUniversidad Complutense de Madrid, Madrid, Spain; 2 Centro de Investigación Biomédica en Red en Enfermedades Cardiovasculares (CIBERCV), Instituto de Salud Carlos III, Madrid, Spain; 3 Hypertension and Vascular Risk Unit, Department of Internal Medicine, Hospital Clínic, University of Barcelona, Barcelona, Spain; 4 Leiria Hospital Centre, Leiria, Portugal; 5 ciTechCare - Center for Innovative Care and Health Technology, Polytechnique of Leiria, Leiria, Portugal; 6 Lipids and Atherosclerosis Unit, IMIBIC/Reina Sofa University Hospital/University of Córdoba, Córdoba, Spain; 7 CIBER Fisiopatologia Obesidad Y Nutricion (CIBEROBN), Instituto de Salud Carlos III, Madrid, Spain

**Correction:** **BMC Proc 17 (Suppl 8), 20 (2023)**

**https://doi.org/10.1186/s12919-023-00268-9**

Following publication of the original article [1], the authors reported that one of the authors listed in the consortia (Multinational Discussion Group) had their incorrect family name published. Adriana Barragan should be Adriana Puente.

The original article [1] has been corrected.
